# The effects of cervical transcutaneous spinal direct current stimulation on motor pathways supplying the upper limb in humans

**DOI:** 10.1371/journal.pone.0172333

**Published:** 2017-02-22

**Authors:** Siobhan C. Dongés, Jessica M. D’Amico, Jane E. Butler, Janet L. Taylor

**Affiliations:** 1 Neuroscience Research Australia, Barker Street, Randwick, New South Wales, Australia; 2 University of New South Wales, Sydney, New South Wales, Australia; Duke University, UNITED STATES

## Abstract

Non-invasive, weak direct current stimulation can induce changes in excitability of underlying neural tissue. Many studies have used transcranial direct current stimulation to induce changes in the brain, however more recently a number of studies have used transcutaneous spinal direct current stimulation to induce changes in the spinal cord. This study further characterises the effects following cervical transcutaneous spinal direct current stimulation on motor pathways supplying the upper limb. In Study 1, on two separate days, participants (n = 12, 5 F) received 20 minutes of either real or sham direct current stimulation at 3 mA through electrodes placed in an anterior-posterior configuration over the neck (anode anterior). Biceps brachii, flexor carpi radialis and first dorsal interosseous responses to transcranial magnetic stimulation (motor evoked potentials) and cervicomedullary stimulation (cervicomedullary motor evoked potentials) were measured before and after real or sham stimulation. In Study 2, on two separate days, participants (n = 12, 7 F) received either real or sham direct current stimulation in the same way as for Study 1. Before and after real or sham stimulation, median nerve stimulation elicited M waves and H reflexes in the flexor carpi radialis. H-reflex recruitment curves and homosynaptic depression of the H reflex were assessed. Results show that the effects of real and sham direct current stimulation did not differ for motor evoked potentials or cervicomedullary motor evoked potentials for any muscle, nor for H-reflex recruitment curve parameters or homosynaptic depression. Cervical transcutaneous spinal direct current stimulation with the parameters described here does not modify motor responses to corticospinal stimulation nor does it modify H reflexes of the upper limb. These results are important for the emerging field of transcutaneous spinal direct current stimulation.

## Introduction

Transcranial direct current stimulation (tDCS) is a well-documented technique involving the application of weak, non-invasive currents to the scalp to induce changes in the excitability of underlying neural tissue (for review see: [1]). Direct current stimulation can also be delivered at the spinal cord level using transcutaneous spinal direct current stimulation (tsDCS) (for reveiws see: [2–4]).

In anaesthetised cat, rat and mouse models, tsDCS can modulate activity in both somatosensory and motor pathways [5–13]. In humans the majority of work has been focused on tsDCS delivered at the thoracic level, with strong evidence to support modifications of ascending lemniscal and nociceptive sensory pathways [14–16] and of spinal reflex pathways including nociceptive reflexes [17, 18], non-nociceptive spinal reflexes elicited by cutaneous afferent stimulation [19] and the H reflex [20–23]. To date, changes reported in the H reflex are consistent with an effect on homosynaptic depression (HD; otherwise known as post activation depression) [21–23], an activity-dependent phenomenon that is thought to occur as a result of reduced release of neurotransmitter from previously activated Ia afferents [24, 25]. Changes in other spinal reflex pathways such as the nociceptive withdrawal reflex [17, 18] are also suggested to occur via modifications to afferents.

Less obvious, is whether or not tsDCS can directly modify descending motor pathways at a spinal level or whether tsDCS modifies the efferent component of spinal reflexes, the α-motoneurones. One study has reported modifications to descending motor pathways after thoracic tsDCS, as measured by a change in lower limb motor evoked potentials (MEPs) elicited using transcranial magnetic stimulation (TMS) [26]. It is unknown where in the motor pathway these changes occur, however there is evidence to suggest that thoracic tsDCS can modulate the excitability of the motor cortex [27].

TsDCS can also be applied to the cervical spinal cord. Cervical tsDCS has been reported to increase upper limb and diaphragm MEPs elicited using TMS [28, 29]. These studies used an anterior-posterior electrode configuration, with the anterior electrode placed midline under the cervicomental angle, and the posterior electrode centred over either the 7^th^ cervical vertebra to increase upper limb MEPs [28] or the 4^th^ cervical vertebra to increase diaphragm MEPs [29]. H reflexes were reported as unchanged after cervical tsDCS [28] but as yet, HD has not been tested.

Changes in MEPs after both thoracic [26] and cervical [28, 29] tsDCS could represent changes at any point along the motor pathway from the motor cortex to the muscles. To determine whether changes occur at a spinal level in the corticospinal pathway after cervical tsDCS, we activated corticospinal axons at the cervicomedullary junction to elicit cervicomedullary MEPs (CMEPs). In addition, because thoracic tsDCS can modify HD of the H reflex in the lower limb, we aimed to determine whether cervical tsDCS could modify HD in the upper limb. In order to compare our findings with previous reports, we also measured MEPs and different parameters of the H-reflex recruitment curve.

## Methods

### Participants

Twelve healthy volunteers (5 F) aged 28 ± 11 years (mean ± SD) completed Study 1, and twelve healthy volunteers (7 F) aged 24 ± 5 years (mean ± SD) completed Study 2 (2 participants completed both studies). Participants were included in Study 1 if they tolerated cervicomedullary stimulation and if CMEPs could be elicited in biceps brachii (4 individuals were excluded out of 16 screened). Participants were included in Study 2 if the H reflex could be clearly observed in FCR at rest, and if the H reflex onset was of a long enough latency to be clearly distinguished from the M wave (8 individuals were excluded out of 20 screened). All participants gave informed, written consent and procedures were approved by the Human Research Ethics Committee of the University of New South Wales. The study was conducted according to the Declaration of Helsinki (2008).

### Transcutaneous spinal Direct Current Stimulation (tsDCS)

Direct current at 3 mA (tDCS stimulator, Transcranial Technologies, Kowloon, Hong Kong) was delivered via two 6 x 5 cm^2^ sponge-covered rubber electrodes soaked in 0.9% saline, giving a current density of 0.1 mA/cm^2^. A modelling study using the same intensity (3 mA) in a different electrode configuration targeting the thoracic spinal cord showed that the maximum current density within the spinal cord (0.085 A/m2) was well below intensities that cause neural damage [30]. Based on the anterior-posterior electrode configuration used in a previous study [28], the anode was placed directly under the cervicomental angle along the midline of the anterior neck, and the cathode was posteriorly centred over the 7^th^ cervical vertebra so that the electrode spanned from C6 to T1. Short axes of the electrodes were placed parallel to the spinal cord. Previous studies using anterior-posterior electrode configurations reveal that the size of muscle responses to brain stimulation increase similarly regardless of the polarity of tsDCS [28, 29]. Thus, we tested cervical tsDCS only with the anode placed anteriorly. Twenty min of either real or sham tsDCS were delivered on two different days in each study. The real tsDCS intervention involved an initial ramping-up of intensity over 30 s, followed by 19.5 min of stimulation at 3 mA. The sham tsDCS intervention involved an initial ramping-up of intensity over 30 s, followed by 1 min of stimulation at 3 mA and then 18.5 min of no stimulation.

### Electromyogram (EMG)

EMG signals were amplified (x 300) and filtered at 16–1000 Hz (CED 1902 amplifier; Cambridge Electronic Design, Cambridge, UK). Data were sampled at 2 kHz, and were recorded on a computer for analysis (CED 1401 with Signal software; Cambridge Electronic Design).

### Study 1

#### Experimental setup

Participants sat upright for the entire experiment with their right arm relaxed on a pillow placed on their lap so the elbow was at ~90°. EMG was recorded from right biceps brachii, flexor carpi radialis (FCR) and first dorsal interosseous (FDI) through Ag-AgCl surface electrodes (20 mm diameter, Conmed, NY, USA) placed over the belly and tendon of each muscle.

#### Brachial plexus stimulation

A constant current stimulator (Model DS7AH, Digitimer, Welwyn Garden City, UK) delivered single stimuli (200 μs pulse width) to peripheral nerves supplying muscles of the right arm through Ag-AgCl surface electrodes (20 mm diameter, Conmed). The cathode was placed in the supraclavicular fossa over the brachial plexus and the anode was placed over the acromion. In order to minimise the total number of stimuli, maximal compound muscle action potentials (M_max_) were elicited in all three muscles (biceps, FCR and FDI) of each participant using the same single stimulus. Stimulus intensity was increased until no further increase in the size of responses from all three muscles occurred, and 120% of this intensity was used (121 ± 46 mA; mean ± SD). M_max_ were elicited throughout the study to monitor for any changes in the muscle fibre action potentials.

#### Cervicomedullary stimulation

Cervicomedullary stimulation was used to activate corticospinal axons subcortically at the cervicomedullary junction. Single electrical pulses (200 μs duration; Digitimer DS7AH) were delivered through Ag-AgCl surface electrodes (20 mm diameter, Conmed) fixed 1-2 cm posterosuperior to the tips of the mastoid processes [31, 32]. Cervicomedullary motor evoked potentials (CMEPs) were recorded from right biceps and FCR muscles. To ensure that responses were elicited by direct corticospinal activation, CMEP onset latency was monitored throughout the study, as a reduction in onset latency of ~2 ms with increasing stimulus intensity represents activation of motoneurones at cervical roots [33]. Biceps CMEP onset latencies of ~ 8 ms and FCR CMEP onset latencies of ~ 10 ms were considered acceptable [32]. Stimulus intensity (134 ± 23 mA; mean ± SD) was set to elicit biceps CMEPs of 1–2 mV (~ 10% M_max_) and consequently FCR responses of ~ 0.5 mV. CMEPs were not reliably evoked in FDI with this stimulus intensity.

#### Transcranial Magnetic Stimulation (TMS)

TMS was used to activate the corticospinal tract at the level of the primary motor cortex. A figure-eight coil (9.5 cm outside loop diameter; Magstim 200, Magstim, Whitland, UK) was positioned over the left motor cortex at the optimal site for preferentially evoking right biceps responses. The optimal site was defined as the position that produced the largest, most consistent responses in the biceps. Although the coil was positioned at the optimal site for eliciting biceps responses, the cortical areas for FCR and FDI muscles are within close proximity, and thus responses in all three muscles can be elicited using the same single stimulus, and any change in the motor pathways supplying each muscle would still be detected. Once found, the site and stimulus intensity was consistent for the duration of each experiment. The coil was placed 45° from midline, handle backwards, to induce a posterior-to-anterior current in the brain. Motor evoked potentials (MEPs) were recorded from right biceps, FCR and FDI. Although our aim was to elicit biceps MEPs of 0.5–1 mV (~5% of M_max_), responses of this size could not be obtained in most participants (11 out of 12), thus biceps MEPs averaged around 1.5% of M_max_. At this stimulus intensity (67 ± 16% of maximum stimulator output; mean ± SD) FCR MEPs of ~ 1 mV and FDI MEPs of ~ 3 mV were obtained.

#### Protocol

A crossover repeated-measures design was implemented, whereby the effects of real and sham cervical tsDCS on motor responses in upper limb muscles were examined on two different days, at least 72 h apart, in pseudorandom order.

**Fig 1:**
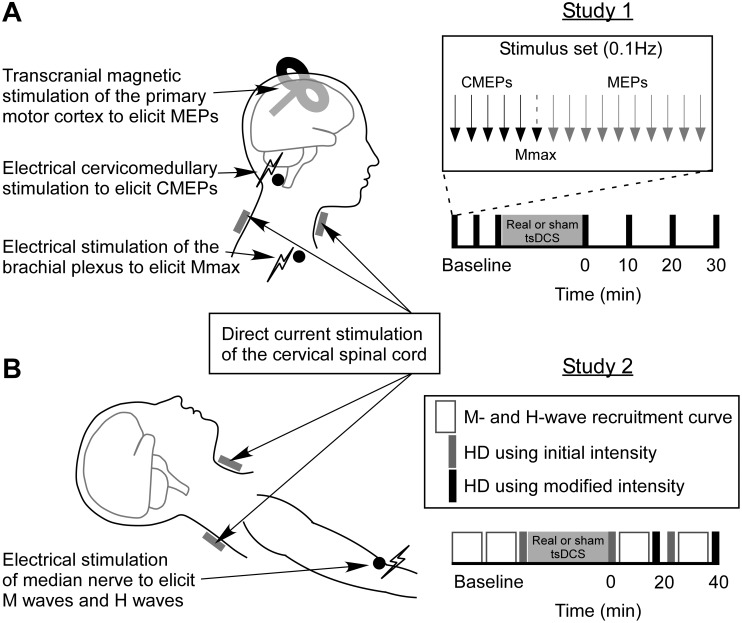
Stimuli and protocols. In Studies 1 and 2, 20 min of real or sham cervical transcutaneous spinal direct current stimulation (tsDCS) was applied through saline soaked sponge electrodes placed anteriorly below the cervicomental angle, and posteriorly over the spinous process of C7. Real tsDCS involved an initial ramping-up of intensity over 30 s, followed by stimulation at 3 mA for the remainder of the 20 min, whereas sham tsDCS involved an initial ramping-up of intensity over 30 s, 1 min of stimulation at 3 mA, then no stimulation for the remainder of the 20 min. In Study 1 **(A)**, participants sat upright with their right arm relaxed on a pillow on their lap. Transcranial magnetic stimulation (TMS) over the primary motor cortex was used to elicit motor evoked potentials (MEPs) in the biceps brachii, flexor carpi radialis (FCR) and first dorsal interosseous (FDI) muscles. Cervicomedullary stimulation was used to directly activate corticospinal axons at the pyramidal decussation, producing cervicomedullary motor evoked potentials (CMEPs) in biceps and FCR. Electrical stimulation of the brachial plexus was used to elicit maximal compound muscle action potentials (M_max_) in the biceps, FCR and FDI. Three baseline sets of stimuli (5 CMEPs, 1 M_max_ and 10 MEPs delivered at 0.1Hz) were delivered prior to real or sham tsDCS, with 5 min between the start of each set. Test sets of stimuli were then delivered at 0, 10, 20 and 30 min after real or sham tsDCS. In Study 2 **(B)**, participants were supine with their right arm out to the side at 45 degrees shoulder abduction, elbow slightly bent, with the palm facing upwards. Electrical median nerve stimulation was used to elicit M waves and H reflexes in the FCR. M-wave and H-reflex recruitment curves were recorded and homosynaptic depression was measured before and after real or sham tsDCS. For baseline measurements, two recruitment curves were recorded, and then one set of stimuli for HD (at intensity for 50% of H_max_). At T1 (0–20 min) and T2 (20–40 min) after real or sham tsDCS, an initial set of stimuli for HD was delivered at the same intensity used for baseline measurements. A recruitment curve was then recorded, and if the stimulus intensity for 50% of H_max_ was different to that measured during baseline recordings, a new set of stimuli for HD was delivered at a modified intensity.

On each day participants underwent a setup protocol for each of the above stimuli (brachial plexus stimulation, cervicomedullary stimulation and TMS). After setup, three sets of baseline stimuli were delivered with 5 min between the start of one set and the next. Each set comprised 16 test stimuli delivered at 0.1 Hz, including 5 CMEPs, 1 M_max_ and 10 MEPs. Real or sham tsDCS was then delivered for 20 min, after which the same stimulus sets (5 CMEPs, 1 M_max_ and 10 MEPs at 0.1 Hz) were delivered at 0, 10, 20 and 30 min after tsDCS offset (Fig 1A).

### Study 2

#### Experimental setup

In Study 2 participants were supine for the entire experiment with their right arm out to the side at 45° shoulder abduction, with the elbow slightly bent and the palm facing upwards [34]. The forearm rested on a cushioned board and was strapped into place. Participants were instructed to stay relaxed and to remain in the same position for the duration of the study. EMG was recorded from the right FCR through Ag-AgCl surface electrodes (20 mm diameter, Conmed) placed over the belly and tendon of the muscle.

#### Median nerve stimulation

A constant current stimulator (Model DS7AH, Digitimer) delivered single stimuli (1 ms pulse width) to the median nerve through Ag-AgCl surface electrodes (20 mm diameter, Conmed). The anode was placed over the median nerve lying medial to the biceps tendon in the cubital fossa. Before placing the cathode, a custom-made, hand-held surface probe was used to locate the median nerve ~2–3 cm proximal to the anode, just medial to the biceps muscle. The optimal location for median nerve stimulation was defined as the position that elicited the largest and clearest H reflexes and M waves in the FCR using the lowest stimulus intensity. Once located, a surface cathode was placed over the median nerve at this site. A constant pressure was maintained over the cathode with a cotton gauze pad held in place with Micropore tape (3M Health Care, Neuss, Germany).

#### H-reflex recruitment curve

H-reflex recruitment curves were recorded by gradually increasing stimulus intensity in steps of either 0.05 or 0.1 mA (2 stimuli per step) from below motor threshold (MT) to above the intensity needed to elicit maximal H reflexes (H_max_). Stimuli were delivered at 0.1 Hz. Increasing the stimuli by 0.05 or 0.1 mA at each step allowed there to be at least 8 points on the ascending portion of the H-reflex recruitment curve for each individual. Once the H-reflex recruitment curve began to descend, intensities were increased in larger steps of 0.5 to 1 mA to obtain the maximal M wave (maximal compound muscle action potential; M_max_) in FCR. Each recruitment curve took ~ 10 min to record.

#### H-reflex homosynaptic depression

HD is strongest when H reflexes are elicited with interstimulus intervals of 1 to 2 s, and at least 10 s between stimuli is needed before HD completely disappears [35]. After applying tsDCS to the thoracic level, Winkler et al. [23] saw bidirectional changes in HD levels depending on the polarity of tsDCS electrodes used, therefore to keep methodology consistent with that of Winkler and colleagues, and to allow for either an increase or decrease in HD levels [36], our stimulus intensity was set so that H reflexes were 50% of H_max_ amplitude. Twenty pairs of stimuli with 1 s interstimulus intervals were delivered to the median nerve each 11 seconds, so that there were 20 H reflexes recorded at a frequency of 1 Hz (H_1Hz_; HD), and 20 H reflexes recorded at 0.1 Hz (H_0.1Hz_; no HD). One set of stimuli for HD took 3.7 min.

#### Protocol

A crossover repeated-measures design was implemented, whereby the effects of real or sham cervical tsDCS on motor responses in upper limb muscles were examined on two different days, at least 72 h apart, in pseudorandom order.

On each day participants underwent a setup protocol for median nerve stimulation. After setup, two baseline recruitment curves were recorded, and then one baseline set of stimuli for HD, using the intensity for 50% of H_max_ as determined from the recruitment curves. Real or sham tsDCS was then delivered for 20 min. After tsDCS, recruitment curves and HD were measured at two time-points (T1: 0–20 min; T2: 20–40 min). At each time-point, an initial set of stimuli for HD was delivered at the same intensity as that used to determine HD in the baseline measurements. A recruitment curve was then recorded, and if the stimulus intensity for 50% of H_max_ was different from that during baseline measurements, an additional set of stimuli for HD was delivered at this modified intensity (Fig 1B). All participants required the use of a modified intensity at least once during the course of the study, possibly due to small changes in arm position that may have resulted in small movements of the stimulating electrodes on the skin relative to the nerve. When this occurred, the level of HD measured at the modified intensity was used for analysis.

### Data analysis and statistics

Root mean squared (RMS) amplitude of EMG was measured for 100 ms prior to stimulation and individual traces were removed from analysis if the biceps, FCR or FDI were not relaxed (~ 2 traces per participant per day). This was to ensure that changes in the size of potentials were not due to differences in muscle activity.

For Study 1, the area under the curve of each CMEP, MEP and M_max_ was measured. To account for any changes in the muscle fibre action potentials over the course of the experiment on a single day, CMEP and MEP areas were normalised to the area of the M_max_ elicited within the same stimulus set. Two-tailed, paired samples t-tests were used to compare the mean of normalised CMEP and MEP baseline measurements on each day of the study (real versus sham tsDCS). Means of 5 CMEPs and 10 MEPs for each stimulus set delivered after real or sham tsDCS were then normalised to the mean of all baseline potentials (15 CMEPs and 30 MEPs).

Two-way repeated measures ANOVAs were used to compare the effects of intervention (real or sham tsDCS) and time (Baseline, 0, 10, 20, and 30 min) on CMEP and MEP areas. Where Mauchly’s test of sphericity was significant, a Greenhouse-Geisser correction was used. One participant was excluded from the MEP analysis due to the presence of a large stimulus artefact which masked the responses.

The study was powered to detect a change in MEPs similar to that previously reported by Lim and Shin in 2011 [28]. From this previous study, FCR MEPs were 32.6 ± 18.8% larger than baseline values immediately after tsDCS using the same anterior-posterior electrode position as that used here, equating to an effect size of 1.73 (Cohen’s d_z_). Nine participants would be sufficient to reveal an effect of this size (two-tails, α = 0.05, power = 0.99) [37]. Here, we had 11 participants for the MEP measure and 12 for the CMEP measure. Statistical significance was set at *p* < 0.05. Group data are presented as means with 95% confidence intervals (CI) unless specified as mean ± SD.

**Fig 2:**
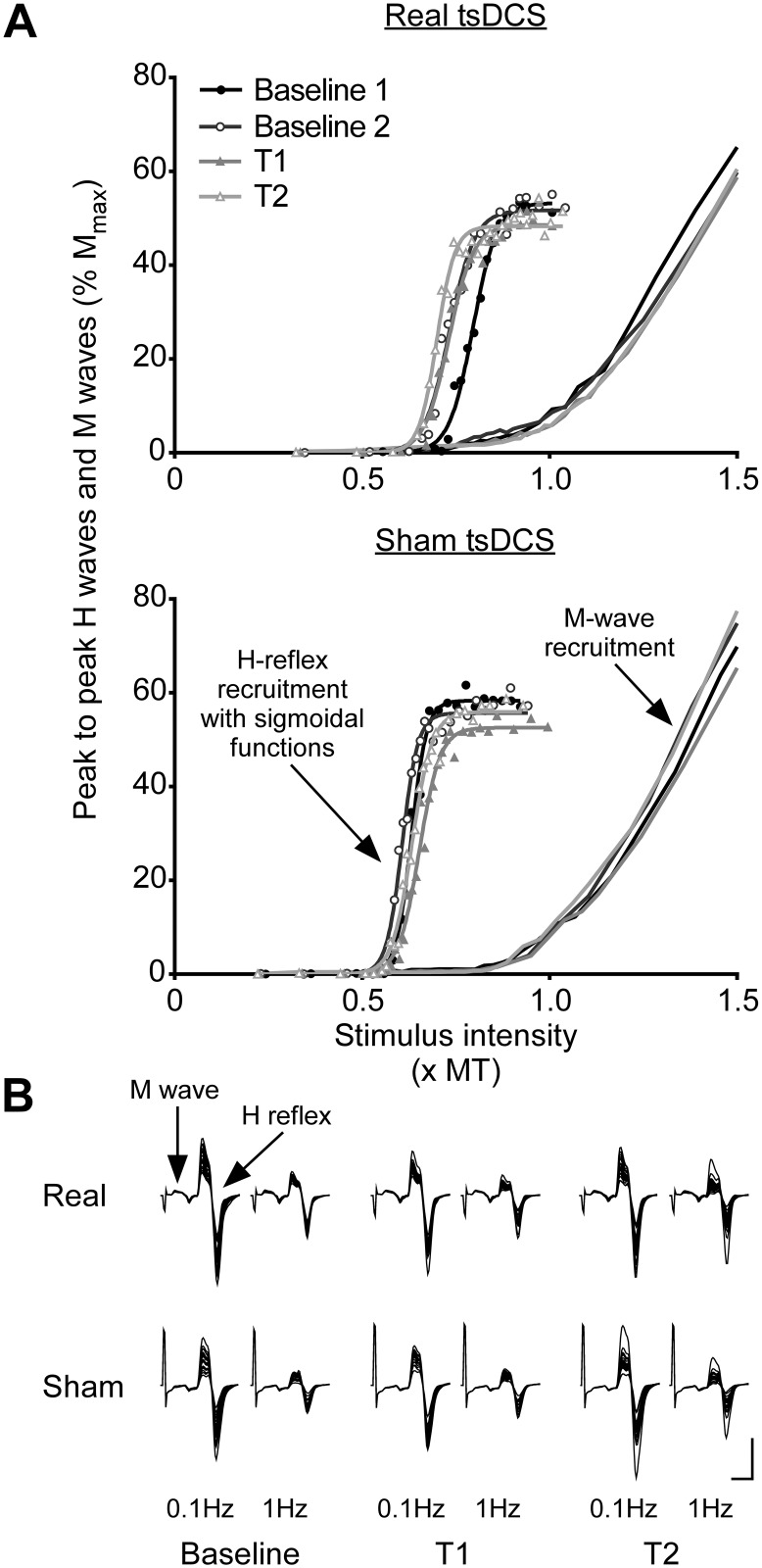
H-reflex recruitment curves, M-wave recruitment curves and H-reflex homosynaptic depression (HD). **(A)** H-reflex and M-wave recruitment curves are for a single participant. H-reflex and M-wave peak-to-peak amplitudes were expressed as a percentage of M_max_. For a better alignment of M-wave recruitment curves, stimulus intensity was normalised to motor threshold (MT). H-reflexes were plotted against stimulus intensity (xMT) until just before the curve began to descend. The three parameter sigmoidal function H(s) = H_max_ / (1 + e^*m*(S50-s)^) was fitted to each H-reflex recruitment curve, where H(s) is the size of the H reflex at a given stimulus intensity (s), S50 is the stimulus intensity required to produce an H reflex of 50% of H_max_, and *m* is the slope parameter of the curve. From sigmoidal functions the following parameters could be calculated: H_max_, slope, S_thresh_ (stimulus intensity to elicit H reflexes of 5% H_max_), S50 and S99 (stimulus intensity to elicit H reflexes of 99% H_max_). In this individual, there are no obvious differences between baseline H-reflex recruitment curves and those measured at T1 (0–20 min) and T2 (20–40 min) after real or sham tsDCS, apart from a slight decrease in H_max_ after both kinds of stimulation. **(B)** Overlaid traces are muscle responses to median nerve stimulation at intensities for eliciting H reflexes of 50% of H_max_. At each time-point (Baseline, T1: 0-20min, T2: 20–40 min), H reflexes were elicited at either 0.1 Hz (no HD) or 1 Hz (HD). Traces are from a different single participant to that in Fig 2A. In this individual there are no obvious differences in the size of H reflexes before and after real or sham tsDCS, nor are there any apparent differences between real or sham tsDCS in the levels of HD. Calibration: vertical, 1 mV; horizontal, 10 ms.

For Study 2, H-reflex and M-wave peak-to-peak amplitudes were measured and normalised to M_max_ and then averaged for each stimulus intensity (2 per average). Averaged and normalised M waves were plotted against stimulus intensity to create M-wave recruitment curves. Motor threshold (MT) was calculated as the *x*-intercept of a straight line fitted to the steepest portion of the curve (for examples see: [38, 39]). To better align M-wave recruitment curves, stimulus intensity was normalised to MT. H-reflex amplitudes were then plotted against normalised stimulus intensity until just before the curve began to descend. Three parameter sigmoidal functions were fitted to the H-reflex recruitment curves using the formula H(s) = H_max_ / (1 + e^*m*(S50-s)^), where H(s) is the size of the H reflex at a given stimulus intensity (s), S50 is the stimulus intensity required to produce an H reflex of 50% of H_max_, and *m* is the slope parameter of the curve (for examples see: [38, 40, 41]). The slope parameter, *m*, reflects the input-output properties of the curve, such that an increase in this parameter indicates a greater change in H-reflex amplitude per unit of stimulus intensity [39,42]. A change in the slope parameter may suggest a change in the overall excitability of the H-reflex pathway. An example of H-reflex recruitment curves with sigmoidal functions and M-wave recruitment curves for a single participant are shown in Fig 2. From the sigmoidal function for each H-reflex recruitment curve the following parameters were calculated: H_max_, slope (defined here as *m*, the slope parameter), S_thresh_ (defined here as the stimulus intensity to elicit H reflexes of 5% H_max_), S50 and S99 (stimulus intensity to elicit H reflexes of 99% H_max_). In order to visualise any group differences in curves, group averages of H_max_, *m* and S50 (taken from equations for each individual’s fitted curves) were used to create three parameter sigmoidal curves.

To provide a measure of the amount of HD, the H_1Hz_ condition was expressed as a percentage of the H_0.1Hz_ condition (H_1Hz_/H_0.1Hz_ x 100) for each stimulus pair. The percentages of HD for 20 pairs were averaged together. For all H-reflex recruitment curve parameters and HD, two-tailed, paired samples t-tests were used to compare baseline measurements on each day of the study (real versus sham tsDCS). All variables were then expressed as a percentage of baseline levels.

Two-way repeated measures ANOVAs were used to compare the effects of intervention (real or sham tsDCS) and time (Baseline, T1 and T2) on MT, levels of HD and on parameters calculated from sigmoidal functions fitted to H-reflex recruitment curves (H_max_, slope, S_thresh_, S50 and S99). Where Mauchly’s test of sphericity was significant, a Greenhouse-Geisser correction was used.

The study was powered to detect a change in HD similar to that previously reported by Winkler et al in 2010 [23]. This previous study revealed a 23.2 ± 14.7% increase in the amount of HD (data extracted from Fig 1B to the nearest 0.005) immediately after thoracic tsDCS (cathode: paravertebral to T11, anode: infraclavicular region) in comparison to baseline. This equates to an effect size of 1.58 (Cohen’s d_z_), and 10 participants would be sufficient to see an effect of this size (two-tails, α = 0.05, power = 0.99) [37]. Here, we had 12 participants for the HD measure. Statistical significance was set at *p* < 0.05. Group data are presented as means with 95% confidence intervals (CI) unless specified as mean ± SD.

## Results

### Study 1

Between days, there were no significant differences in baseline CMEPs or MEPs elicited prior to the delivery of real or sham tsDCS for any muscle (Table 1; *p* > 0.05).

**Table 1:** Mean baseline values for CMEPs, MEPs, H-reflex recruitment curve parameters and HD show no significant differences between days.

	Real tsDCS day	Sham tsDCS day	p*
**CMEP (% M**_**max**_**)**			
Biceps	9.46 (6.44 to 12.5)	9.07 (6.60 to 11.5)	0.64
FCR	3.53 (1.43 to 5.64)	1.89 (1.27 to 2.51)	0.05
**MEP (% M**_**max**_**)**			
Biceps	1.36 (0.364 to 2.36)	1.25 (0.404 to 2.09)	0.61
FCR	5.33 (2.89 to 7.76)	7.16 (2.84 to 11.5)	0.14
FDI	15.6 (5.54 to 25.6)	17.1 (6.91 to 27.3)	0.08
**Recruitment curve parameters**			
H_max_ (% M_max_)	29.6 (20.6 to 38.5)	25.6 (16.1 to 35.1)	0.19
Slope	25.1 (22.3 to 28.0)	28.1 (19.9 to 36.4)	0.44
Sthresh (x MT)	0.730 (0.664 to 0.797)	0.726 (0.661 to 0.790)	0.87
S50 (x MT)	0.859 [0.798 to 0.919)	0.874 (0.782 to 0.966)	0.72
S99 (x MT)	1.06 (0.999 to 1.12)	1.11 (0.908 to 1.30)	0.61
**HD**			
H_1Hz_ (% H_0.1Hz_)	43.8 (33.0 to 54.6)	44.1 (36.7 to 51.6)	0.93

Data are presented as mean values with lower and upper limit 95% CIs.

* *p* values were produced with two-tailed, paired samples t-tests comparing baseline values on each day of the study.

**Fig 3:**
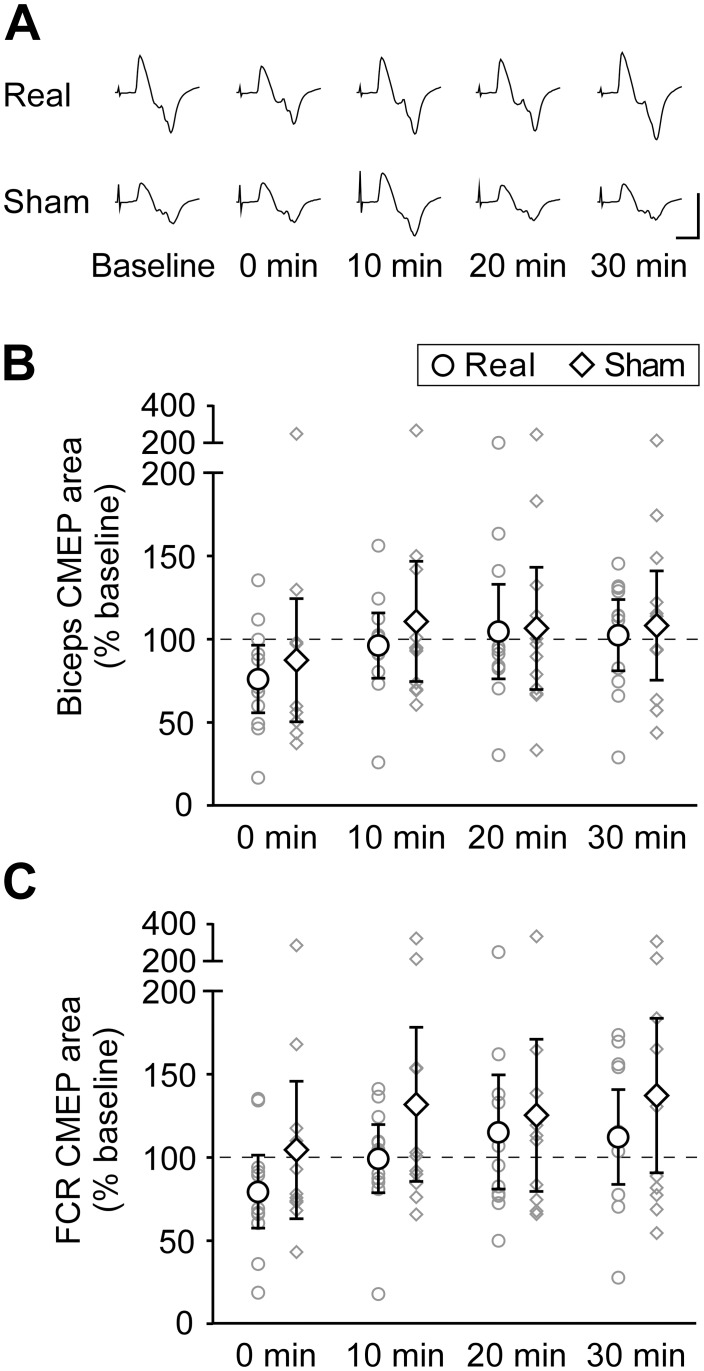
Muscle responses to stimulation at the cervicomedullary junction before and after real or sham tsDCS. **(A)** Averaged biceps brachii CMEPs (5–15 traces) for a single participant elicited before (Baseline) and at 0, 10, 20, and 30 min after real or sham tsDCS. No major differences can be observed between responses measured before and after real or sham interventions. Calibration: vertical, 1 mV; horizontal, 10 ms. Biceps **(B)** and FCR **(C)** CMEP areas are normalised to M_max_ and expressed as a percentage of baseline values. Both group means (n = 12; **◯** = tsDCS; **◇** = sham) and individual data (smaller grey symbols) are represented at 0, 10, 20 and 30 min after real or sham tsDCS. Error bars are 95% CIs. For both biceps and FCR, there are no significant changes in CMEP size over time, and there are no significant differences between real and sham tsDCS.

Cervical tsDCS had no effect on CMEPs in any muscle. The area of biceps CMEPs did not change over time (main effect of time: *F*_(2.12,23.35)_ = 2.37, *p* = 0.11), nor was there any difference between real and sham interventions (main effect of intervention: *F*_(1,11)_ = 0.61, *p* = 0.45). There was no interaction effect of time and intervention on CMEP area (*F*_(4,44)_ = 0.46, *p* = 0.77). Similarly for FCR, there was no effect of time (*F*_(1.89,20.80)_ = 3.23, *p* = 0.06) or intervention (*F*_(1,11)_ = 2.21, *p* = 0.17) and no interaction effect (*F*_(1.93,21.18)_ = 1.73, *p* = 0.20) on the area of CMEPs. Fig 3 shows group means of biceps and FCR CMEPs (n = 12), as well as biceps CMEP traces from a single individual before and after real and sham tsDCS.

**Fig 4:**
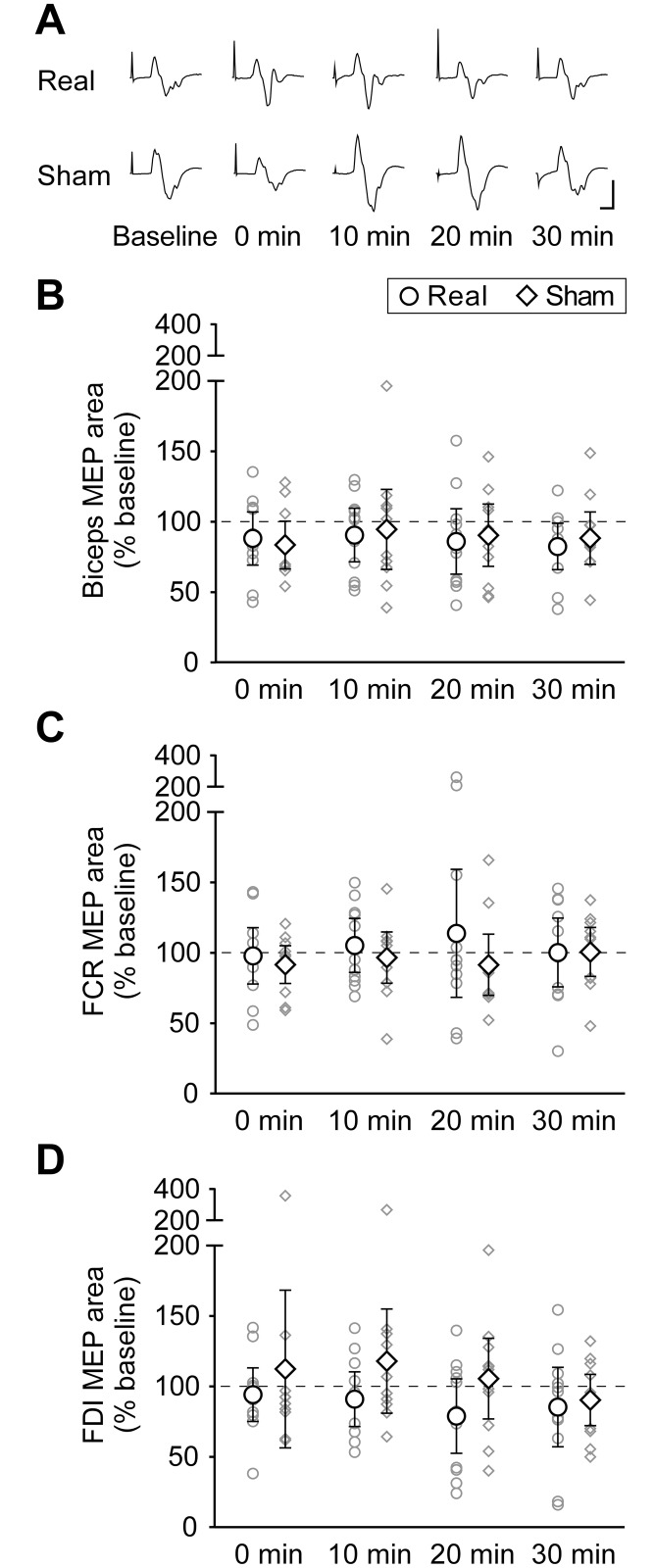
Muscle responses to transcranial magnetic stimulation of the primary motor cortex before and after real or sham tsDCS. **(A)** Averaged biceps brachii MEPs (10–30 traces) for a single participant elicited before (Baseline) and at 0, 10, 20, and 30 min after real or sham tsDCS. No major differences can be observed between responses measured before and after real or sham interventions. Calibration: vertical, 0.1 mV; horizontal, 10 ms. Biceps **(B)**, FCR **(C)** and FDI **(D)** MEP areas are normalised to M_max_ and expressed as a percentage of baseline values. Both group means (n = 11; **◯** = tsDCS; **◇** = sham) and individual data (smaller grey symbols) are represented at 0, 10, 20 and 30 min after real or sham tsDCS. Error bars are 95% CIs. For all three muscles, there are no significant changes in MEP size over time, and there are no significant differences between real and sham interventions.

Similarly to CMEPs, MEPs displayed no changes after either real or sham tsDCS. There was no effect of time on any muscle (biceps: *F*_(4,40)_ = 1.22, *p* = 0.32; FCR: *F*_(4,40)_ = 0.25, *p* = 0.91; FDI: *F*_(2.02,20.16)_ = 0.88, *p* = 0.43), nor an effect of intervention (biceps: *F*_(1,10)_ = 0.06, *p* = 0.81; FCR: *F*_(1,10)_ = 0.66, *p* = 0.44; FDI: *F*_(1,10)_ = 1.67, *p* = 0.23). There was no interaction of time and intervention (biceps: *F*_(4,40)_ = 0.22, *p* = 0.93; FCR: *F*_(1.63,16.34)_ = 0.60, *p* = 0.53; FDI: *F*_(1.67,16.69)_ = 0.71, *p* = 0.48). Fig 4 shows group means of biceps, FCR and FDI MEPs (n = 11), as well as biceps MEP traces from a single individual before and after real and sham tsDCS.

### Study 2

There were no differences in baseline levels of any of the H-reflex recruitment curve parameters between real and sham tsDCS days (Table 1; *p* > 0.1), and there was no difference in MT between or within real and sham tsDCS days as measured using the x-intercept method of the M-wave recruitment curve (main effect of time: *F*_(1.11,12.26)_ = 0.18, *p* = 0.71; main effect of intervention: *F*_(1,11)_ = 0.18, *p* = 0.68; interaction of time and intervention: *F*_(1.17,12.81)_ = 0.21, *p* = 0.69).

There was a significant decrease in H_max_ over time for both real and sham interventions (main effect of time: *F*_(1.33,14.64)_ = 10.66, *p* = 0.003), but there was no difference between real and sham tsDCS (main effect of intervention: *F*_(1,11)_ = 0.14, *p* = 0.72), and there was no interaction of time and intervention (*F*_(2,22)_ = 0.18, *p* = 0.84). Similarly, there was a significant decrease in the slope parameter over time for both interventions (main effect of time: *F*_(2,22)_ = 4.49, *p* = 0.02), but no difference between real and sham tsDCS (main effect of intervention: *F*_(1,11)_ = 0.40, *p* = 0.54) or an interaction between intervention and time (*F*_(2,22)_ = 0.13, *p* = 0.88). For the remaining H-reflex recruitment curve parameters (S_thresh_, S50, S99), there were no significant differences over time (S_thresh_: *F*_(1.22,13.45)_ = 1.41, *p* = 0.27; S50: *F*_(2,22)_ = 0.89, *p* = 0.42; S99: *F*_(1.31,14.35)_ = 2.26, *p* = 0.15), no differences between real and sham tsDCS (S_thresh_: *F*_(1,11)_ = 3.00, *p* = 0.11; S50: *F*_(1,11)_ = 0.02, *p* = 0.90; S99: *F*_(1,11)_ = 1.66, *p* = 0.22), and no interactions between time and intervention (S_thresh_: *F*_(1.12,12.27)_ = 0.53, *p* = 0.50; S50: *F*_(2,22)_ = 0.02, *p* = 0.98; S99: *F*_(2,22)_ = 0.43, *p* = 0.66). To summarise, although there were decreases in both H_max_ and the slope of the H-reflex recruitment curve over time, there were no differences between real and sham tsDCS, and no interactions between time and intervention for any of the H-reflex parameters measured from FCR.

**Fig 5:**
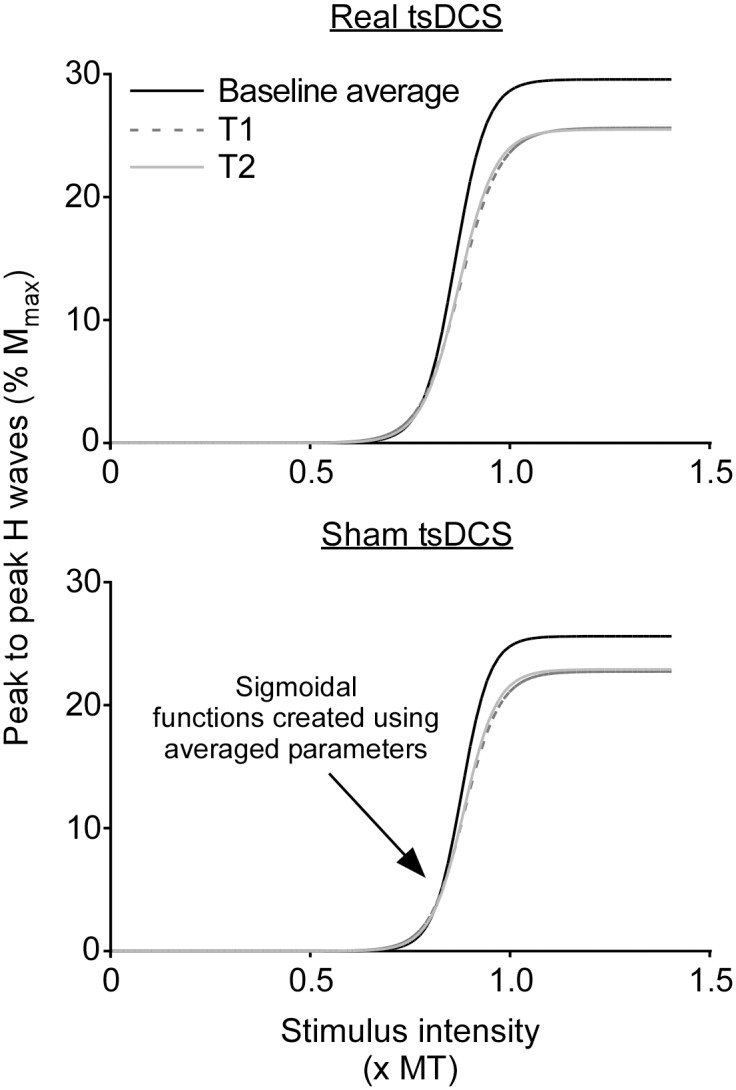
Sigmoidal functions using group data. In order to visualise any differences in H-reflex recruitment curves, the group averages (n = 12) of H_max_, *m* and S50 (taken from equations for each individual’s fitted curves) were used to create three parameter sigmoidal curves using the formula H(s) = H_max_ / (1 + e^*m*(S50-s)^). From these curves a small difference in baseline H_max_ between real and sham tsDCS days can be observed, however this difference is not significant (Table 1). Also of note is the decrease in H_max_ and slope of the curves at both T1 (0–20 min) and T2 (20–40 min) after tsDCS in comparison to baseline, however these differences occurred similarly on both real and sham tsDCS days, as can be seen in Fig 6A and 6B.

**Fig 6:**
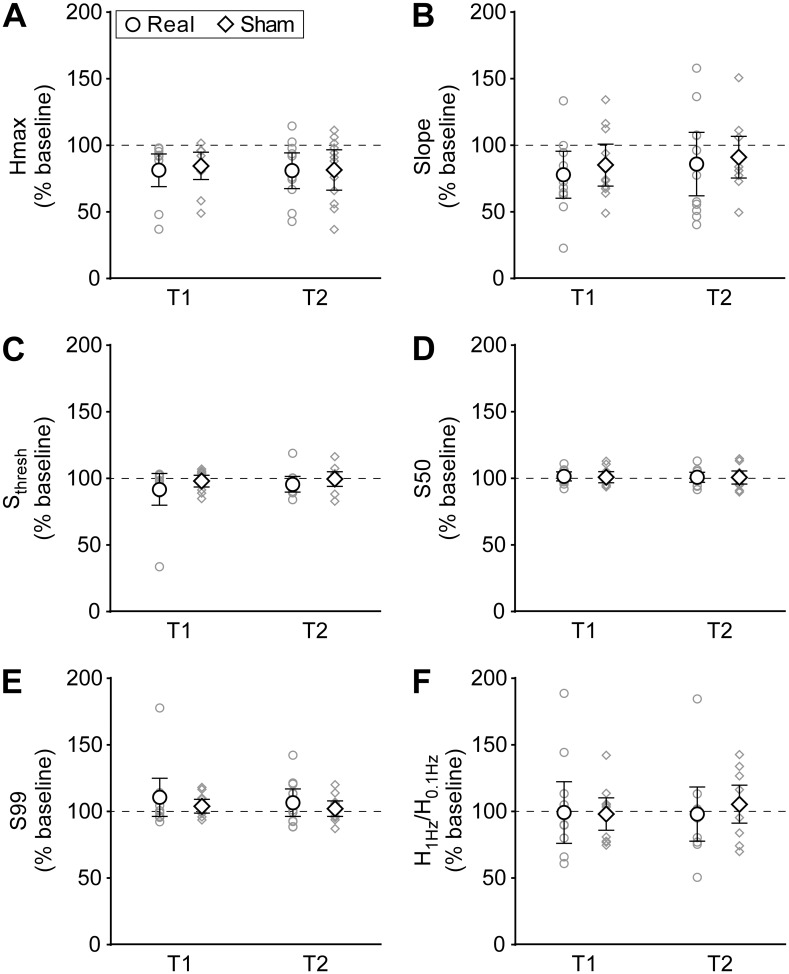
Variables of the H-reflex recruitment curve and homosynaptic depression (HD) of the H reflex. Both group means (n = 12; **◯** = tsDCS; **◇** = sham) and individual data (smaller grey symbols) are represented at T1 (0–20 min) and T2 (20–40 min) after real or sham tsDCS. Error bars are 95% CIs. Both H_max_ **(A)** and slope **(B)** of the H-reflex recruitment curve are decreased in comparison to baseline measurements, however there are no differences between real and sham tsDCS, with similar decreases seen after both kinds of stimulation. For the remaining H-reflex recruitment curve parameters, Sthresh **(C)**, S50 **(D)** and S99 **(E)**, there are no differences before and after stimulation and there are no differences between real and sham tsDCS. There are similarly no differences in HD of the H reflex, calculated using H_1Hz_/H_0.1Hz_ **(F)**. Note that values are given as a % of baseline; thus a value greater than 100% indicates less HD and a value less than 100% indicates more HD than baseline.

There were no differences in baseline levels of HD between real and sham tsDCS days (Table 1; *p* = 0.93). Levels of HD did not differ over time (*F*_(2,22)_ = 0.17, *p* = 0.84) and there was no difference between real and sham tsDCS (*F*_(1,11)_ = 0.09, *p* = 0.77), or an interaction between time and intervention (*F*_(2,22)_ = 0.38, *p* = 0.69). Fig 2 shows H-reflex and M-wave recruitment curves, as well as raw traces for HD in an individual. Fig 5 shows sigmoidal functions created using group averages of H-reflex recruitment curve parameters and Fig 6 shows group means and individual data for all H-reflex recruitment curve parameters and for HD.

## Discussion

Results presented here suggest that the application of 20 minutes of cervical tsDCS at 3 mA using an anterior-posterior electrode configuration does not modify upper limb muscle responses to either TMS or cervicomedullary stimulation, nor does it modify any of the tested parameters of the H-reflex recruitment curve or HD within 30 min after stimulation. This may suggest that cervical tsDCS using this set of stimulation parameters does not modify descending motor pathways at cortical, spinal or motoneuronal levels, nor does it modify afferent or efferent components of the H-reflex pathway.

Previous studies showed facilitation of MEPs after cervical tsDCS applied using an anterior-posterior electrode configuration [28, 29]. As increases in MEPs can occur through increases in cortical excitability or through changes occurring at a spinal level, we investigated whether CMEPs were altered by tsDCS. CMEPs are generated by activation of corticospinal axons at the level of the cervicomedullary junction and hence, are insensitive to changes in cortical excitability but are altered by changes in motoneurone excitability or in the efficacy of corticospinal transmission in the spinal cord. To compare our findings with those from previous reports we also used TMS to elicit MEPs. Here we found no change in CMEPs after cervical tsDCS, suggesting that there is no modification to the corticospinal pathway at the spinal level. In contrast to previous findings, we also saw no change in MEPs after cervical tsDCS, suggesting that descending motor pathways were also not modified at a supraspinal level [28, 29]. It is important to note that due to the large size of surface recording electrodes (20 mm diameter), crosstalk from nearby muscles could have contributed to the recorded responses, particularly with the smaller muscles, FCR and FDI. However, the motoneurones of neighbouring muscles are anatomically close and should be affected similarly by tsDCS. Therefore, despite the possibility of crosstalk, the results suggest that cervical tsDCS using the parameters described here does not modify responses in elbow flexors, wrist flexors or distal hand muscles.

The parameters used in our study were primarily based on those used by Lim and Shin, who delivered 20 min of 2 mA tsDCS through 25 cm^2^ electrodes placed in the same anterior-posterior configuration as that used here [28]. FCR MEPs were reported as facilitated for 2 hours after delivering tsDCS at 2 mA, where we saw no change using 3 mA. Although current density reaching the spinal cord during cervical tsDCS has not been modelled, there are individual differences in current density that actually reach the spinal cord during thoracic tsDCS [30]. Given that such individual differences can exist, it is unlikely that the differences in stimulus intensity or electrode size explain the difference between observing facilitation and observing no change. However, a tDCS study reported a shift from inhibitory to excitatory modulation of the human motor cortex when the intensity was increased from 1 to 2 mA [43]. Thus it may be of benefit to repeat the current study using 2 mA of tsDCS through 25-cm^2^ electrodes to determine whether the precise stimulus intensity and electrode size are critical.

In another study, cervical tsDCS applied using an anterior-posterior electrode configuration resulted in an increase in the size of diaphragm MEPs after stimulation regardless of the polarity of electrodes [29]. This study used 15 min of 2.5 mA tsDCS delivered via 35 cm^2^ electrodes placed on the anterior neck under the chin and the posterior neck over spinal segments C3-C5. Despite an overall similarity in stimulation protocols, there are differences between this study and ours that may explain the differences in MEP results. Most obviously, the diaphragm is phasically active during breathing, and would therefore be active during tsDCS application, whereas for our study the tested muscles were at rest for the duration of the study. In line with this, there is evidence to suggest that combining tDCS of the motor cortex with a motor task improves performance of that task [44]. However there are also studies showing that only tDCS delivered before, but not during motor training is effective at increasing MEP size [45], and voluntary muscle activity during tDCS can reverse the changes to motor cortical excitability [46]. Given these contrasting results, it is difficult to predict the effect of muscle activity during cervical tsDCS, however this is a key difference between the current study and that of Niérat and colleagues [29]. Interactions between tsDCS and muscle activity warrant further investigation.

Cervicomedullary stimulation causes some brief discomfort, due to the activation of local skin afferents underneath each electrode [33]. As pain has been shown to modulate the excitability of descending motor pathways [47–49], there is a possibility that these painful stimuli may have had an effect on the induction of plasticity in Study 1. Studies describing the effects of pain on plasticity induction show mixed results. Tonic cutaneous pain increases the corticospinal excitability induced by ischaemia [50], whereas tonic intra-oral pain during a tongue motor training task blocks the induction of motor cortical plasticity [51] and local tonic pain during a finger movement training task does not affect plasticity [52]. Given these mixed results, it is difficult to predict the effects of brief, painful electrical stimuli on tsDCS. However, brief, painful laser stimuli only induce changes in motor cortical excitability that last up to 150 ms [53]. Therefore, it is unlikely that the similarly brief CMEPs, which were not elicited during tsDCS, would have an effect that lasted long enough to interfere with the tsDCS. Furthermore, previous studies have used CMEPs to demonstrate that spinal-level plasticity can be induced using paired stimulus protocols [54–56]. Hence, the delivery of cervicomedullary stimulation does not preclude the induction of plasticity at a spinal level.

Thoracic tsDCS using a monopolar electrode configuration (one posteriorly centred over T9-T11 and one over the right shoulder) can modify properties of MEPs depending on the polarity of stimulating electrodes [26]. When the anode is placed over the spinal cord, MEP resting motor threshold is increased, whereas when the cathode is placed over the spinal cord, the size of MEPs is increased. Although this study uses thoracic tsDCS instead of cervical tsDCS, it suggests that a monopolar electrode configuration may be better for eliciting modifications to descending motor pathways. Furthermore, the direction of electrical field with respect to the orientation of neurons is critical to the effects of DCS [57, 58]. Thus it is possible that a more longitudinal configuration of electrodes may be more effective at modifying motor pathways supplying the upper limb.

To determine whether cervical tsDCS could modify Ia afferents or α-motoneurones, we assessed H-reflex recruitment curves and homosynaptic depression of the H reflex. There were no changes in the stimulus intensities required to produce H reflexes of 5% (S_thresh_), 50% (S50) and 99% (S99) of H_max_; and although there were decreases in both H_max_ and slope of the H-reflex recruitment curve over time, there were no differences between real and sham tsDCS. Our H-reflex data confirm previous work in the upper and lower limbs which reported no change in threshold intensity, maximal H-reflex size and the intensity required for a maximal H reflex [26, 28] and hence, suggest no changes in motoneurone excitability. Although a shift in the H-reflex recruitment curve is reported after thoracic tsDCS, these changes only occurred when stimuli were delivered at intervals that result in HD [22], whereas when recruitment curves were created using stimuli at longer intervals, no changes were seen in their properties [17]. On the other hand, we did not observe any changes in the amount of HD of H reflexes, contrary to previous work done in the lower limb, which showed that thoracic tsDCS can modify HD in a bidirectional manner, depending on electrode polarity [23]. Thus, the data presented here suggest that cervical tsDCS with anterior-posterior electrode positioning does not influence subsequent motoneurone excitability, nor does it alter the behaviour of Ia afferent terminals in the way described for thoracic tsDCS using a monopolar electrode configuration.

Despite the study being sufficiently powered to detect changes similar to those previously reported, the variability within our data appears greater than that in previous studies. Thus, it is possible that true small changes in the various outcome measures have not been detected because of lack of power. However examination of the confidence intervals and the spread of data in Figs 3, 4 and 6, with some individuals increasing and some decreasing on both real and sham tsDCS days, suggests that it is unlikely that an increase in sample size would reveal an important effect.

In the emerging field of tsDCS it is of vital importance that both negative and positive results are published, so that collaboratively, the methodology can be optimised, and the true potential of the technique realised. Negative results are often not submitted for publication, or if they are submitted, they are less likely to be published or cited [59–61]. Although our data are negative findings, they contribute to this newly developing field by revealing a particular set of stimulus parameters that was not able to induce modifications to descending motor pathways or H reflexes in this sample. The results suggest that an anterior-posterior electrode configuration is not optimal for tsDCS. A monopolar or more longitudinal configuration of electrodes, or voluntary contraction of the tested muscle during tsDCS may be more effective for modification of motor pathways.

## Supporting information

S1 dataset Dataset for study 1. (XLSX)

S2 dataset Dataset for study 2. (XLSX)
